# Dynamic risk prediction of BK polyomavirus reactivation after renal transplantation

**DOI:** 10.3389/fimmu.2022.971531

**Published:** 2022-08-17

**Authors:** Yiling Fang, Chengfeng Zhang, Yuchen Wang, Zhiyin Yu, Zhouting Wu, Yi Zhou, Ziyan Yan, Jia Luo, Renfei Xia, Wenli Zeng, Wenfeng Deng, Jian Xu, Zheng Chen, Yun Miao

**Affiliations:** ^1^ Department of Transplantation, Nanfang Hospital, Southern Medical University, Guangzhou, China; ^2^ Department of Biostatistics, School of Public Health (Guangdong Provincial Key Laboratory of Tropical Disease Research), Southern Medical University, Guangzhou, China

**Keywords:** BK polyomavirus, reactivation, dynamic Cox regression, prediction, renal transplantation

## Abstract

**Purpose:**

To construct a dynamic prediction model for BK polyomavirus (BKV) reactivation during the early period after renal transplantation and to provide a statistical basis for the identification of and intervention for high-risk populations.

**Methods:**

A retrospective study of 312 first renal allograft recipients with strictly punctual follow-ups was conducted between January 2015 and March 2022. The covariates were screened using univariable time-dependent Cox regression, and those with P<0.1 were included in the dynamic and static analyses. We constructed a prediction model for BKV reactivation from 2.5 to 8.5 months after renal transplantation using dynamic Cox regression based on the landmarking method and evaluated its performance using the area under the curve (AUC) value and Brier score. Monte-Carlo cross-validation was done to avoid overfitting. The above evaluation and validation process were repeated in the static model (Cox regression model) to compare the performance. Two patients were presented to illustrate the application of the dynamic model.

**Results:**

We constructed a dynamic prediction model with 18 covariates that could predict the probability of BKV reactivation from 2.5 to 8.5 months after renal transplantation. Elder age, basiliximab combined with cyclophosphamide for immune induction, acute graft rejection, higher body mass index, estimated glomerular filtration rate, urinary protein level, urinary leukocyte level, and blood neutrophil count were positively correlated with BKV reactivation, whereas male sex, higher serum albumin level, and platelet count served as protective factors. The AUC value and Brier score of the static model were 0.64 and 0.14, respectively, whereas those of the dynamic model were 0.79 ± 0.05 and 0.08 ± 0.01, respectively. In the cross-validation, the AUC values of the static and dynamic models decreased to 0.63 and 0.70 ± 0.03, respectively, whereas the Brier score changed to 0.11 and 0.09 ± 0.01, respectively.

**Conclusion:**

Dynamic Cox regression based on the landmarking method is effective in the assessment of the risk of BKV reactivation in the early period after renal transplantation and serves as a guide for clinical intervention.

## Introduction

BK polyomavirus (BKV) is an important factor that threatens graft function in renal transplant recipients, leading to allograft failure and even urothelial carcinoma (1). Recently, the increasing number of secondary transplantations, highly sensitized patients, and long-term administration of potent immunosuppressants have resulted in a higher rate of BKV reactivation. The incidence of BKV viruria and viremia among recipients has reached 23%–73% and 8%–62%, respectively (2, 3). Without clinical symptoms, the diagnosis of BKV reactivation relies on quantitative polymerase chain reaction (PCR) of BKV DNA in body fluids during regular follow-up (4). Consequently, the detection rate of BKV is closely related to the follow-up schedule, focus on BKV detection, and medical level of follow-up centers. Owing to the lack of specific antiviral therapies for BKV viruria and viremia as well as BKV allograft nephropathy (BKVAN), we can only reduce immunosuppressant use or use immunoglobulin and cidofovir to control increased viral titers due to BKV reactivation (5, 6). Batal et al. reported that early intervention when urine BKV was positive contributed to the low rate of long-term graft loss, but therapy initiated when urine BKV was high was not effective and had an unsatisfactory prognosis (7). Therefore, determination of the main risks and protective factors, identification of high-risk populations, and early systemic interventions for prognosis improvement are important tertiary prevention strategies for BKV reactivation.

Several studies have explored the relevant static factors of BKV replication in renal transplant recipients with inconsistent results (8–16); in particular, the effect of immunosuppressive regimens on BKV replication remains controversial. For example, rapamycin (mTOR) inhibitors could inhibit BKV replication, whereas tacrolimus has the opposite effect, according to the research of Hirsch et al. (14). However, a systematic review of 28 randomized, controlled trials did not confirm a reduction of BKV infection with an mTOR inhibitor-based regimen compared with a calcineurin inhibitor-based regimen (15). Because most of the current studies regard BKV viremia or even BKVAN as the endpoint for risk factor analysis, a gap in knowledge remains in the early stage of BKV reactivation. However, the possibility of the effects of various factors being time-dependent is undeniable. For instance, Hirsch et al. indicated that steroids were a risk factor of BKV reactivation during the first 3 months posttransplantation, whereas tacrolimus, older age, and male sex contributed to later reactivation at 12 months (10). Therefore, to understand the influence of dynamic indices, the risk factors for BKV reactivation after renal transplantation with consideration of time should be analyzed, and a dynamic prediction model for the risk should be constructed. The landmarking method is commonly used for dynamic prediction. By selecting the restricted sample of the data at a specific moment in time to form landmark datasets, this method enables the comprehensive analysis of the baseline information and follow-up data for the risk prediction (17).

On the basis of covariates selected by univariable time-dependent Cox regression, the present study constructed prediction models for BKV reactivation from 2.5 to 8.5 months after renal transplantation using static and dynamic Cox regression based on the landmarking method, respectively, thus providing a statistical basis for the identification and intervention of high-risk populations for BKV reactivation.

## Materials and methods

### Cohort

We retrospectively analyzed 312 first renal allograft recipients who kept strictly punctual follow-ups (break of follow-ups no more than twice a year and delay no more than a week) at Nanfang Hospital (Guangzhou, China) between January 2015 and March 2022. The study was approved by the Ethics Committee of Nanfang Hospital, Southern Medical University (NFEC-2020-044), and was conducted according to the Declaration of Helsinki guidelines. Owing to the retrospective nature of the study, the requirement for the informed consent was waived. Patients with plasma or urine BKV DNA ≥5000 copies/mL before renal transplantation, with plasma or urine BKV DNA ≥5000 copies/mL within 15 days after transplantation, without regular follow-up records after transplantation, without tacrolimus and mycophenolic acid as the maintenance regimen, with no records of plasma or urine BKV DNA levels during follow-up, with BKV viremia (plasma BKV DNA ≥5000 copies/mL) at the point of BKV reactivation, or graft failure during follow-up were excluded. The enrolled recipients were divided into two groups according to urine BKV DNA status: BKV-positive (urine BKV DNA ≥5000 copies/mL) and BKV-negative groups.

### Sample processing

Urine and peripheral EDTA-blood samples were collected for the detection of BKV DNA load by quantitative real-time PCR using BKV nucleic acid detection kit (Sinomd Gene, Beijing, China) and the fully automatic medical PCR analysis system (SLAN^®^-96P; Sansure Biotech, Hunan, China). The primers BKV-F and BKV-R in the PCR process were AGAACTGCTCCTCAATGGATG and AGCTGCCCCTGGACACTCT, respectively.

Human cytomegalovirus (CMV) DNA load was detected using a CMV detection kit (ZJ Bio-Tech Co., Ltd., Shanghai, China) and a fluorescence quantitative PCR instrument (Cobas Z 480; Roche, Basel, Switzerland). The primers CMV-F and CMV-R in the PCR process were GAAGGTGCAGGTGCCCTG and GTGTCGACGAACGACGTACG, respectively.

The estimated glomerular filtration rate (eGFR) was calculated using the serum creatinine-based Chronic Kidney Disease Epidemiology Collaboration formula.

### Data collection

The following clinical information was collected: static baseline data, including sex, age, body mass index (BMI), dialysis time, induction protocol, and delayed graft function (DGF); clinical events and dynamic indices related to BKV reactivation, including acute rejection (AR), CMV infection, eGFR, uric acid (UA), blood leukocyte count (WBC), blood neutrophil count (NE), blood lymphocyte count (LYM), blood monocyte count (MON), hemoglobin (HGB), platelet count (PLT), serum albumin (ALB), blood glucose (GLU), blood tacrolimus concentration (Tac), blood mycophenolic acid concentration (MPA), urinary leukocyte (uWBC), urinary erythrocyte (uRBC), and urinary protein (uPRO). Urinary erythrocyte, leukocyte and protein level was divided into negative (-), low level (±,1+) and high level (2+,3+ and 4+).

### Diagnostic criteria of CMV infection

Diagnosis of CMV infection depends on symptoms and signs, and the gold standard is a CMV DNA load >500 copies/mL in the blood, urine, or bronchoalveolar lavage fluid (18).

### Diagnostic criteria of DGF

Diagnosis of DGF is based on the presence of at least one of the following criteria: undergoing at least one hemodialysis during the first postoperative week; serum creatinine level >400 µmol/L on postoperative day 10; serum creatinine level >300 µmol/L on postoperative day 14 (19, 20). The increased serum creatinine levels resulting from early AR and nephrotoxicity of immunosuppressive agents should be excluded first.

### Diagnostic criteria of AR

Diagnosis of AR is based on the occurrence of typical clinical manifestations, including unexplained reduction of urine volume, increased serum creatinine, graft swelling, and tenderness. Color Doppler ultrasonography of the graft indicates an increase in vascular resistance index (>0.75) of all levels of arteries without vascular or ureteral complications. The results of graft biopsy conform to the histologic criteria for diagnosing AR according to Banff guidelines (21). Because of the limitation due to the retrospective design of the study, some recipients were diagnosed based on clinical manifestations and color Doppler ultrasonography by excluding other possibilities but without histopathological examination.

### Analysis of baseline variables

The baseline variables were analyzed using SPSS version 23.0 (IBM Corp., Armonk, NY, USA). Normally distributed variables are reported as means ± standard deviation, and non-normal variables are reported as medians (minimum–maximum). Categorical variables are reported as numbers with percentages. Comparisons of classified variables between the BKV-positive and BKV-negative groups were performed using chi-square test. For continuous variables with variance homogeneity using Levene’s test, comparisons were performed through independent-sample t-test. For continuous variables not satisfying variance homogeneity, Mann-Whitney U test was used for comparison.

### Variable screening

Considering that the collected data were longitudinal, variable screening was performed using univariable time-dependent Cox regression. The cutoff value was 0.1, and the selected covariates were included in the subsequent static and dynamic analyses.

### Construction of a static model

Based on the covariates selected by univariable time-dependent Cox regression, backward selection was used to select the covariates according to the Akaike information criterion (AIC) (22). A static Cox regression model was constructed based on the baseline data 15 days after renal transplantation. The performance of the prediction model was then evaluated by discrimination (area under the curve, AUC) and calibration (Brier score).

### Construction of a dynamic model

**Figure 1:**
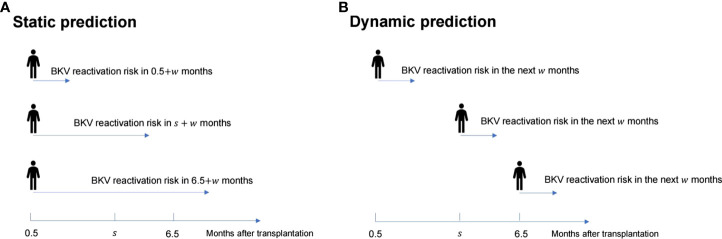
Schematic diagram of the difference between the static prediction **(A)** and dynamic prediction **(B)** processes.

Dynamic analysis was performed using a dynamic Cox regression based on the landmark approach. For the dynamic Cox regression model, based on the follow-up data from 0.5 to 6.5 months, the prediction time points were chosen from every month; {*s_i_*, *i* = 0,…, *L*} = {*s*_0_, *s_i_*, …, *s*_6_} = {0.5, 1.5, …, 6.5} were used to predict the next *w* months, which indicates the probability of BKV reactivation from 0.5 + *w* to 6.5 + *w* months (**Figure 1**). For each *s_i_*, the corresponding landmark dataset *R_i_* was constructed, which included the follow-up population in *s_i_* - *s_i_* + *w* months. The follow-up was restricted to *s_i_* + *w* month, which means that the time for patients without BKV reactivation changed to *s_i_* + *w* month, ignoring the ending event. For numerical stability, the prediction time was standardized using *s* / (*s_L_* – *s*_0_).

All landmark datasets were stacked into a single “super prediction dataset” and fitted with a Cox model. The effects of covariates, an interaction term for covariates and predicted time $\bar{s}$ , interaction term for covariates and quadratic term of the predicted time ${\bar{s}}^{2}$ on the positive rate of BKV were evaluated (23). According to the AIC, covariates with P<0.1 in univariate time-dependent Cox regression were screened using backward selection. The regression coefficients of covariates, covariates and $\bar{s}$ , covariates, and ${\bar{s}}^{2}$ were reported, and the changes in the hazard ratio (HR) of covariates with $\bar{s}$ are presented in **Figure 1** (24). The dynamic prediction model was performed by R code, which is available in Supplementary File S1.

### Evaluation and validation of dynamic model performance

The fitted dynamic Cox model was used to predict the outcomes of each *s_i_*, which were then compared with the actual data to obtain the performance measures (AUC and Brier score). The baseline static model was also used to predict the outcome, and the AUC and Brier score were calculated.

Monte-Carlo cross-validation of models was done to avoid overfitting. Specifically, the data were divided into a train set (80% samples) and a test set (20% samples). The train set was used to refit the above models, which were tested in the left samples to obtain another group of performance measures (AUC and Brier score). The entire process was repeated 100 times, providing 100 groups of AUCs and Brier scores. The average of these measurements at each time point represents the performance of the models in the cross-validation.

The personal prediction was performed using the dynamic Cox model, which means that the probability of BKV reactivation in the next *w* months after the postoperative *s* months could be predicted.

All statistical tests were performed at a two-sided significance level of 0.05, and all modeling analyses were performed using R version 4.0.2 (The R Foundation, Vienna, Austria).

## Results

### Baseline statistics

A total of 112 patients were excluded, and finally, 200 recipients were enrolled, which were classified as BKV-positive (121 cases) or BKV-negative (79 cases). **Table 1** shows the patients’ clinical characteristics and demographic data.

**Table 1:** The baseline clinical characteristics and demographic data.

Variates	BKV positive (n=121)	BKV negative (n=79)	P value
**Sex (n,%)**			0.682
Male	86, 71.07%	54, 68.35%	
Female	35, 28.93%	25, 31.65%	
**Age (year)**	42.70 ± 11.72	41.32 ± 12.95	0.436
**BMI (kg/m^2^)**	22.25 ± 3.61	21.32 ± 3.46	0.074
**Induction (n,%)**			0.000
Basiliximab	11, 9.09%	28, 35.44%	
ATG	23, 19.01%	16, 20.25%	
Basiliximab+ATG	83, 68.60%	32, 40.51%	
Basiliximab+ cyclophosphamide	3, 2.48%	3, 3.80%	
ATG+ rituximab	1, 0.82%	0, 0.00%	
**Dialysis modality (n, %)**			0.288
Hemodialysis	81, 66.94%	51, 64.56%	
Peritoneal dialysis	21, 17.36%	21, 26.58%	
Alternation	7, 5.78%	3, 3.80%	
NA	12, 9.92%	4, 5.06%	
**Dialysis time (month)**	7.20 (0–125.93)	9.67 (0–135.27)	0.096
**DGF (n, %)**			0.997
Yes	23, 19.01%	15, 18.99%	
No	98, 80.99%	64, 81.01%	

BMI, body mass index, ATG, antithymocyte globulin, NA, no dialysis, DGF, delayed graft function.

The analysis of sex, induction, dialysis modality and DGF were performed using chi-square testing. The analysis of age,BMI were performed using independent-sample t testing while the analysis of dialysis time was performed using Mann-Whitney U testing.

**Figure 2:**
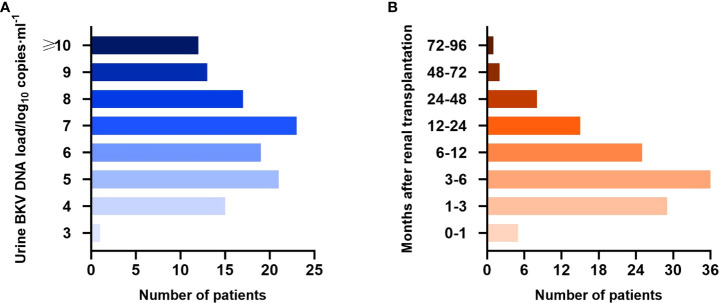
Urine BK polyomavirus (BKV) DNA load **(A)** and time distribution of urine BKV DNA positivity for the first time **(B)** in the BKV-positive group.

Until the first time that BKV-positive patients were positive for urine BKV DNA and the last time that BKV-negative patients were negative, the median follow-up period was 178.5 (22–2183) days posttransplantation. Specifically, the median follow-up of BKV-positive patients was 159 (22–2183) days and that of BKV-negative patients was 225 (25–2083) days. The load and time distribution of urine BKV DNA positivity for the first time are presented in **Figures 2A, B**, respectively.

### Univariate time-dependent Cox regression

**Table 2** shows the univariate time-dependent Cox regression of the potentially covariates, among which covariates with P<0.1 were included in the static and dynamic analyses.

**Table 2:** Results of univariable time-dependent Cox regression.

Variates	HR	SE	P
sex (ref: female)	1.213	0.056	0.001
age	1.004	0.002	0.053
BMI	1.031	0.007	0.000
induction	0.718	0.034	0.000
dialysis	0.997	0.001	0.008
DGF	1.340	0.058	0.000
AR	1.610	0.061	0.000
CMV	0.633	0.279	0.101
eGFR	0.976	0.010	0.011
UA	0.999	0.002	0.834
ALB	0.799	0.031	0.000
WBC	1.002	0.009	0.858
NE	1.021	0.010	0.050
LYM	0.986	0.003	0.000
MON	0.995	0.011	0.642
HGB	0.984	0.013	0.240
PLT	0.981	0.004	0.000
GLU	1.024	0.018	0.193
Tac	1.044	0.008	0.000
MPA	1.002	0.009	0.857
uWBC	1.104	0.054	0.065
uRBC	1.178	0.039	0.000
uPRO	1.222	0.053	0.000

BMI, body mass index; induction, immune induction scheme; dialysis, dialysis time; DGF, delayed graft function; AR, acute rejection; CMV, cytomegalovirus infection; eGFR, estimated glomerular filtration rate; UA, uric acid; ALB, serum albumin; WBC, white blood cell; NE, blood neutrophil count; LYM, blood lymphocyte count; MON, blood monocyte count; HGB, hemoglobin; PLT, platelet count; GLU, blood glucose; Tac, blood tacrolimus concentration; MPA, blood mycophenolic acid concentration; uWBC, urinary leukocyte; uRBC,urinary erythrocyte; uPRO, urinary protein; HR, hazard ratio; SE, standard error.

### Static analysis and model evaluation

**Figure 3:**
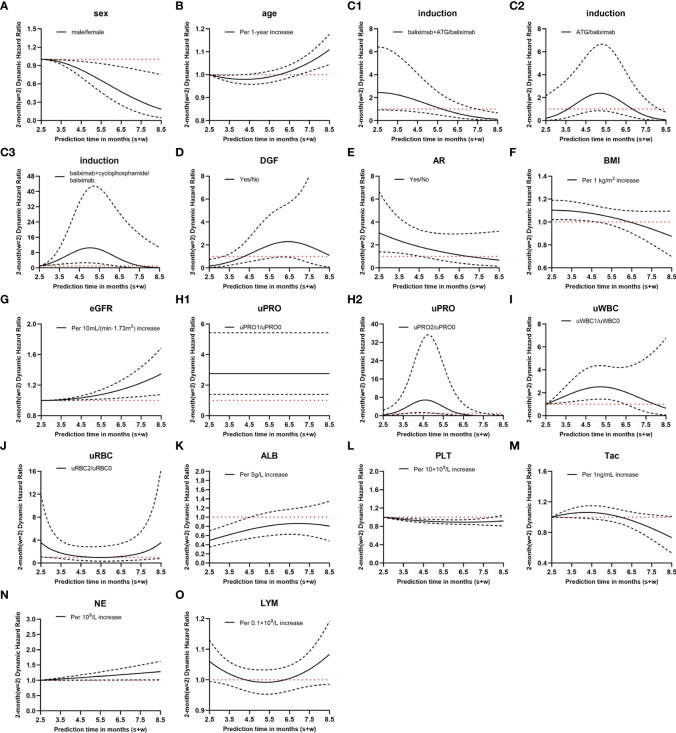
Hazard ratio (HR) values of dynamic Cox regression. **(A–O)**, different variates; C1–3, different immune induction schemes; H1–2, different levels of urinary protein; solid lines, dynamic HR of different variates; dashed lines, 95% confidence intervals; red dotted lines, HR=1; induction, immune induction scheme; ATG, antithymocyte globulin; DGF, delayed graft function; AR, acute rejection; BMI, body mass index; eGFR, estimated glomerular filtration rate; uPRO, urinary protein; uPRO0, negative result of urinary protein; uPRO1, low level of urinary protein; uPRO2, high level of urinary protein; uWBC, urinary leukocyte; uWBC0, negative result of urinary leukocyte; uWBC1, low level of urinary leukocyte; uRBC, urinary erythrocyte; uRBC0, negative result of urinary erythrocyte; uRBC2, high level of urinary erythrocyte; ALB, serum albumin; PLT, platelet count; Tac, blood tacrolimus concentration; NE, blood neutrophil count; LYM, blood lymphocyte count.

Postoperative day 15 was set as the baseline, and variables with P<0.1 were analyzed using stepwise regression. The static prediction model for BKV reactivation was established based on the baseline data, and the final model included the variables presented in **Table 3** and **Figure 3**. Data showed that acute rejection, eGFR, low level of urinary leukocyte (compared with a negative result), and serum albumin exerted predictive significance for endpoint events, and the first three of which were risk factors, whereas serum albumin was a protective factor for BKV reactivation.

**Table 3:** Results of static Cox regression model.

Variates	HR	SE	P
AR	2.067	0.281	0.010
eGFR [per 10mL/(min·1.73m^2^)]	1.097	0.036	0.011
ALB (per 5 g/L)	0.759	0.105	0.009
uWBC1(ref: uWBC0)	1.416	0.196	0.076

AR, acute rejection; eGFR, estimated glomerular filtration rate; ALB, serum albumin; uWBC0, negative result of urinary leukocyte; uWBC1, low level of urinary leukocyte; HR, hazard ratio; SE, standard error.

**Figure 4:**
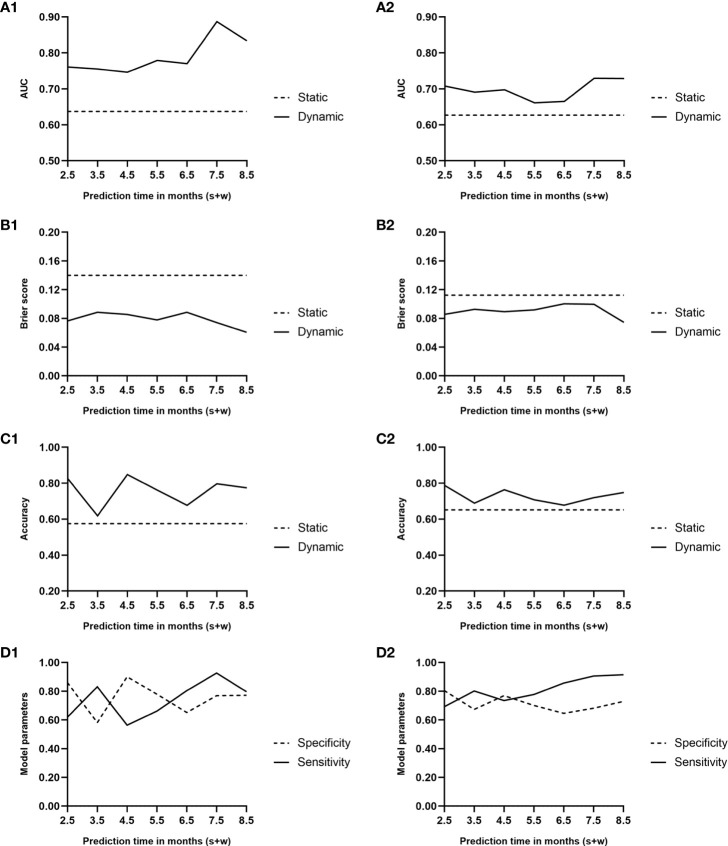
Area under the curve **(A1, A2)**, Brier score curve **(B1, B2)**, and accuracy curve **(C1, C2)** during the predicted period of the two models. Solid lines, dynamic model; dashed lines, static model. **(D1, D2)** Sensitivity and specificity curves during the predicted period of the dynamic model. Solid line, sensitivity; dashed line, specificity; 1, performance of the two models; 2, results of the Monte Carlo cross-validation.

The AUC and Brier score were 0.64 and 0.14, respectively, and the accuracy of the static model reached 0.58 (**Figures 4A1, B1, C1**). In the cross-validation, the AUC and Brier score were 0.63 and 0.11, respectively, whereas the accuracy was 0.65 (**Figures 4A2, B2, C2**).

### Dynamic analysis and model evaluation

Considering the clinical significance, we made a comparative analysis with *w* = 1, 2, and 3. Since the model had the best performance when *w* = 2, only the modeling results when *w* = 2 are presented here. **Supplementary Table S1** shows the covariates most related to BKV reactivation screened by stepwise regression and the regression coefficient for interaction terms 1, $\bar{s}$ , and ${\bar{s}}^{2}$ . The relationship between the HR value of the covariates and follow-up time is shown in **Figure 3**. For instance, compared with basiliximab, the HR of antithymocyte globulin (ATG) was $\text{HR}\left(\bar{s}\right)=\text{exp}\left(-1.585+10.866\;\;\times \;\bar{s}-12.024\;\;\times \;{\bar{s}}^{2}\right)$ . Assuming $\bar{s}=0.5$ , at 3.5 months after transplantation, the HR of recipients using ATG for immune induction was *e* ^−1.585+10.866 ×0.5−12.024 × 0.5^2^^ ~2.32 times higher than those using basiliximab in the following 2 months.

The final risk score of BKV reactivation 2.5–8.5 months after renal transplantation:


$$\begin{array}{llllllllllllll} PI=-1.688\;\times (sex\\ =male)\times {\bar{s}}^{2}-0.143\times age\times \bar{s}+0.246\times \;age\times {\bar{s}}^{2}+0.894 & \times \left(induction=baliximab+ATG\right)-3.089\times \left(induction=baliximab+ATG\right) & \times \;{\bar{s}}^{2}-1.585\times \left(induction=ATG\right)+10.866\times \left(induction=ATG\right)\times \bar{s}-12.024 & \times \;\left(induction=ATG\right)\times {\bar{s}}^{2}+11.186\;\times \;(induction=baliximab+cyclophosphamide) & \times \;\bar{s}-13.25\times \left(induction=baliximab+cyclophosphamide\right) & \times \;{\bar{s}}^{2}-1.838\times \left(DGF=Yes\right)+8.134\times \left(DGF=Yes\right) & \times \;\bar{s}-6.202\times \left(DGF=Yes\right)\times {\bar{s}}^{2}+1.116\times \left(AR=Yes\right)-1.498\times \left(AR=Yes\right) & \times \;\bar{s}+0.098\times BMI\left(kg/m^{2}\;\right)-0.232\times BMI\left(kg/m^{2}\;\right)\times {\bar{s}}^{2}+0.298\times eGFR\left[10mL/\left(min\cdot 1.73m^{2}\;\right)\right] & \times \;{\bar{s}}^{2}+1.014\times \left(uPRO=uPRO1\right)-1.105\times \left(uPRO=uPRO2\right)+17.336\times \left(uPRO=uPRO2\right) & \times \;\bar{s}-24.756\times \left(uPRO=uPRO2\right)\times {\bar{s}}^{2}+4.075\times \left(uWBC=uWBC1\right)\times \bar{s}-4.493\times \left(uWBC=uWBC1\right) & \times \;{\bar{s}}^{2}+1.269\times \left(uRBC=uRBC2\right)-5.142\times \left(uRBC=uRBC2\right)\times \bar{s}+5.151\times (uRBC=uRBC2) & \times \;{\bar{s}}^{2}-0.701\times ALB\left(5g/L\right)+1.496\times ALB\left(5g/L\right)\times \bar{s}-1.01\times ALB\left(5g/L\right)\times {\bar{s}}^{2}-0.344\times PLT\left(10\times 10^{9}/L\right) & \times \;\bar{s}+0.258\times PLT\left(10\times 10^{9}/L\right)\times {\bar{s}}^{2}+0.422\times Tac\left(ng/mL\right)\times \bar{s}-0.733\times Tac\left(ng/mL\right)\times {\bar{s}}^{2}+0.25\times NE\left(10^{9}/L\right) & \times \bar{s}+0.058\times LYM\left(0.1\times 10^{9}/L\right)-0.287\times LYM\left(0.1\times 10^{9}/L\right)\times \bar{s}+0.309\times LYM\left(0.1\times 10^{9}/L\right)\times {\bar{s}}^{2} \end{array}$$

The dynamic model revealed that during the follow-up period of 0.5–6.5 months after renal transplantation, the variables that maintained a significant positive correlation with BKV reactivation throughout were eGFR, uPRO1 (ref:uPRO0), and NE, whereas male sex remained a protective factor.

**Figures 4A1, B1** presents the trends of AUC and Brier score, respectively, over time in the dynamic model. The average AUC was 0.79 ± 0.05. In comparison, the average AUC of the model when *w* = 1 and *w* = 3 were 0.79 ± 0.06 and 0.76 ± 0.04, respectively. During the follow-up period of 0.5–6.5 months after renal transplantation, the performance of the dynamic model was superior to that of the static model, which was based on the baseline all along.

Based on the AUC value at every predicted moment, the cutoff value of the risk score was obtained at the maximum Youden index. A risk score exceeding the cutoff value indicates a higher risk of BKV reactivation in the following month. The accuracy of the dynamic model along with specificity and sensitivity are shown in **Figures 4C1, D1**.

**Figures 4A2, B2, C2, D2** illustrates the results of the cross-validation of the dynamic model. The average AUC was 0.70 ± 0.03. In comparison, the average AUCs of the model when *w* = 1 and *w* = 3 were 0.67 ± 0.07 and 0.66 ± 0.03, respectively. Although the AUC and accuracy curves decreased while Brier score increased on the whole, the performance was still superior to that of the static one.

### Individual dynamic prediction

**Figure 5:**
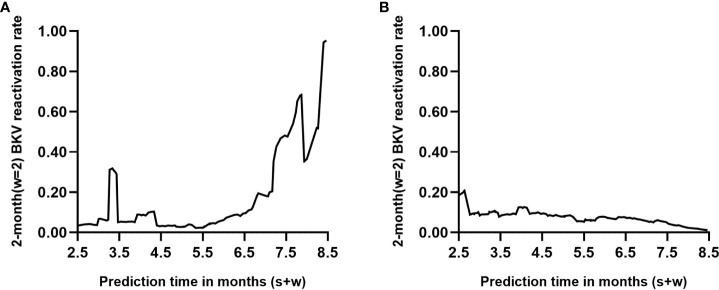
Individual prediction with the dynamic model (s=6, w=2). Each point on the curve refers to the probability of BK polyomavirus (BKV) reactivation in month s+2 based on follow-up records within s months posttransplant. **(A, B)** represent two renal transplant recipients of known endpoint events who turned out to be positive and negative within 8.5 months posttransplantation, respectively.

Two renal transplant recipients of known endpoint events were selected from the dataset (see Supplementary Data Sheets for details). Recipient A developed urine BKV DNA at 6.7 months posttransplant, whereas recipient B remained negative for urine BKV DNA at follow-up at 8.5 months. **Figure 5** shows the dynamic occurrence rate of BKV reactivation in the future 2 months. According to the real-life condition of the two recipients, the performance of the dynamic model can be determined.

## Discussion

BKV reactivation is highly prevalent in renal transplant recipients, mainly occurring within 1 year after transplantation, with a peak period of 3–6 months (10). Here, we designed follow-up strategies according to the Kidney Disease: Improving Global Outcomes guideline 2009 (18), that is, detecting plasma and urine BKV DNA loads once a month within 3–6 months after the renal transplantation and every 3 months in the following 6 months. The temporal epidemiological distribution of the BKV reactivation population in our center is consistent with those in other domestic and foreign centers.

Cox regression is the common means to predict the risk of endpoint events. However, due to perioperative trauma, graft implantation, high-dose hormone, and immunosuppressive agents, the homeostasis of immune, circulatory, endocrine, and other systems reconstructs with biochemical and physical indices varying with the external disturbance. The static prediction model based on baseline or some fixed-point during follow-up neglects the time dependency of certain covariates (23). In this way, the predicted probability cannot update with the changes in personal conditions. Hence, we proposed a dynamic prediction model based on baseline information and the regular detection indices during the postoperative 0.5–6.5 months. The postoperative 2.5–8.5 months was designed as a predictive time, and the following 2 months were the predictive time window of BKV reactivation. When compared with the static model, the trends in the early-stage physical changes and the individual heterogeneity of the renal transplant recipients were shown to be fully considered in the dynamic model. In addition, the role of the effective factors was reinforced, and the internal and external disturbances were reduced, thus achieving high sensitivity and specificity with an average AUC value up to 0.79 and accuracy up to 0.76. Subsequently, the model was validated using Monte-Carlo cross-validation. Similar to the above results, the performance in the cross-validation indicated the superiority of the dynamic model over the static one.

We determined the related risk factors for early BKV reactivation using the dynamic model, and the results indicated that the weight of these factors varied at different time points. Cyclophosphamide, a potent immunosuppressive agent, is commonly used for immune induction in highly sensitized recipients and for treating recurrent glomerulonephritis after renal transplantation (25). Our study indicated that, unlike the stable dose alleviating autoimmune reactions in the chronic kidney disease stage (26), cyclophosphamide shock treatment showed a remarkable promoting effect on BKV reactivation in highly sensitized recipients during the first 2.5–6 months. In addition, the role of acute rejection and positive urinary protein in BKV reactivation is precise (27), presenting a trend similar to that of cyclophosphamide. This might be attributed to HLA mismatch (28) and medically increasing immunosuppression after then. Urinary tract infection with positive urinary leukocyte and excessive blood neutrophils counts and other opportunistic pathogen infection are also risk factors for BKV reactivation, which indicates hypoimmunity. Obesity was proven to lead to chronic low inflammation, immune system disorders, and impaired defensive function, thus increasing the risk of infection (29, 30). Similarly, a higher BMI showed significant facilitation of BKV reactivation during the postoperative 2.5–4.5 months in our study. The serum albumin level reflects hepatic synthesis function, nutritional status, and inflammation and serves as an indicator of the progression of acute and chronic wasting diseases. A low level of serum albumin could influence the stability of globin and could indicate an exhausted status (31). Some studies have shown that hypoalbuminemia contributes to increased infection by opportunistic pathogens, such as CMV and BKV, after renal transplantation (32). Our results revealed a protective effect of serum albumin on BKV reactivation.

The limitation of this study lies in the single-center retrospective design. Because of different donor sources, quality of donor grafts, and matching rules in different centers, donor factors and HLA matching factors were not included in the independent variables in order to improve the universality of the model, whereas related indirect variables such as occurrence of DGF and acute rejection and creatinine fluctuation trend after surgery were included as a remedy.

Based on the dynamic model, we provided a valuable statistical experience for the prevention of early BKV reactivation after renal transplantation. Compared with the existing static model for BKV reactivation (16), there is a noticeable progress in discrimination (AUC value), which is from 0.69 to 0.79. Additionally, the model emphasized a point of view that post-transplant management is not merely the management of the graft; on the contrary, it is comprehensive management containing multiple factors and systems. Patients cannot extend the follow-up interval randomly, even with good graft function, and clinicians are not expected to pursue rapid recovery of graft function, but rather to ignore the potential risks. The application of the dynamic model is beneficial for precise risk stratification of BKV reactivation and for achieving a balance between rejection and antiviral immunity. Early screening and intervention prevent the incidence of BKV infection-related diseases in high-risk populations. Furthermore, the development of individualized monitoring and treatment prevents the waste of healthcare resources to achieve maximum economic and social benefits.

## Data availability statement

The raw data supporting the conclusions of this article will be made available by the authors, without undue reservation.

## Author contributions

YW and YM designed and directed the study. YF, YW, and YZ wrote the manuscript. YF, ZW, ZYYa, JL, RX, WZ, and WD were responsible for data collection and check. YF, CZ, and ZYYu performed data analysis and validation. YLF took charge for data visualization. JX, YM, and ZC revised the manuscript. All authors read and approved the final version.

## Funding

This study was funded by the National Natural Science Foundation of China (grant nos. 82070770 and 82173622), the Natural Science Foundation of Guangdong Province (grant nos. 2020A1515010674, 2022A1515011525, and 2019A1515011506), and the National College Students’ Innovative Entrepreneurial Training Plan Program (grant nos. 202212121024 and 202212121242).

## Conflict of interest

The authors declare that the research was conducted in the absence of any commercial or financial relationships that could be construed as a potential conflict of interest.

## Publisher’s note

All claims expressed in this article are solely those of the authors and do not necessarily represent those of their affiliated organizations, or those of the publisher, the editors and the reviewers. Any product that may be evaluated in this article, or claim that may be made by its manufacturer, is not guaranteed or endorsed by the publisher.
