# Corrigendum: Dynamic risk prediction of BK polyomavirus reactivation after renal transplantation

**DOI:** 10.3389/fimmu.2023.1325584

**Published:** 2023-11-14

**Authors:** Yiling Fang, Chengfeng Zhang, Yuchen Wang, Zhiyin Yu, Zhouting Wu, Yi Zhou, Ziyan Yan, Jia Luo, Renfei Xia, Wenli Zeng, Wenfeng Deng, Jian Xu, Zheng Chen, Yun Miao

**Affiliations:** ^1^ Department of Transplantation, Nanfang Hospital, Southern Medical University, Guangzhou, China; ^2^ Department of Biostatistics, School of Public Health (Guangdong Provincial Key Laboratory of Tropical Disease Research), Southern Medical University, Guangzhou, China

**Keywords:** BK polyomavirus, reactivation, dynamic Cox regression, prediction, renal transplantation

In the published article, there was an error. In the description of cohort screening, an important inclusion criterion was not mentioned, which is “with strictly punctual follow-ups”, based on modeling requirements. It does not affect the actual studied cohort which has already followed the requirements.

A correction has been made to **Abstract: Methods**. This sentence previously stated:

“A retrospective study of 312 first renal allograft recipients was conducted between January 2015 and March 2022”

The corrected sentence appears below:

“A retrospective study of 312 first renal allograft recipients with strictly punctual follow-ups was conducted between January 2015 and March 2022.”

A correction has been made to **Materials and Methods**, *Cohort*. This sentence previously stated:

“We retrospectively analyzed 312 first renal allograft recipients at Nanfang Hospital (Guangzhou, China) between January 2015 and March 2022.”

The corrected sentence appears below:

“We retrospectively analyzed 312 first renal allograft recipients who kept strictly punctual follow-ups (break of follow-ups no more than twice a year and delay no more than a week) at Nanfang Hospital (Guangzhou, China) between January 2015 and March 2022.”

The authors apologize for these errors and state that this does not change the scientific conclusions of the article in any way. The original article has been updated.

