# Supplementary material for: Dynamic risk prediction of BK polyomavirus reactivation after renal transplantation
Source: Front Immunol. 2022 Aug 17;13:971531. doi: 10.3389/fimmu.2022.971531 (PMC9428263; doi:10.3389/fimmu.2022.971531)
Supplement: Supplementary file 1 [file DataSheet_1.zip › Supplementary Table 1.docx]

**Supplementary Table S1. Results of dynamic Cox regression model when *w=2***

| Variates | Time | Coefficient | SE | P |
| --- | --- | --- | --- | --- |
| **sex(ref: female)** | $\bar{s}^{2}$ | -1.688 | 0.715 | 0.018 |
| **age** | $\bar{s}$ | -0.143 | 0.057 | 0.012 |
|  | $\bar{s}^{2}$ | 0.246 | 0.076 | 0.001 |
| **induction(ref: basiliximab)** |  |  |  |  |
| basiliximab+ATG | 1 | 0.894 | 0.493 | 0.070 |
|  | $\bar{s}^{2}$ | -3.089 | 1.090 | 0.005 |
| ATG | 1 | -1.585 | 1.204 | 0.188 |
|  | $\bar{s}$ | 10.866 | 4.633 | 0.019 |
|  | $\bar{s}^{2}$ | -12.024 | 4.328 | 0.005 |
| basiliximab+cyclophosphamide | $\bar{s}$ | 11.168 | 3.328 | 0.001 |
|  | $\bar{s}^{2}$ | -13.25 | 4.85 | 0.006 |
| **DGF(ref: No)** | 1 | -1.838 | 0.787 | 0.020 |
|  | $\bar{s}$ | 8.134 | 3.887 | 0.036 |
|  | $\bar{s}^{2}$ | -6.202 | 4.234 | 0.143 |
| **AR(ref: No)** | 1 | 1.116 | 0.396 | 0.005 |
|  | $\bar{s}$ | -1.498 | 0.997 | 0.133 |
| **BMI** | 1 | 0.098 | 0.039 | 0.012 |
|  | $\bar{s}^{2}$ | -0.232 | 0.131 | 0.078 |
| **eGFR** | $\bar{s}^{2}$ | 0.298 | 0.114 | 0.009 |
| **uPRO(ref: uPRO0)** |  |  |  |  |
| uPRO1 | 1 | 1.014 | 0.346 | 0.003 |
| uPRO2 | 1 | -1.105 | 1.016 | 0.277 |
|  | $\bar{s}$ | 17.336 | 7.05 | 0.014 |
|  | $\bar{s}^{2}$ | -24.756 | 9.151 | 0.007 |
| **uWBC(ref: uWBC0)** |  |  |  |  |
| uWBC1 | $\bar{s}$ | 4.075 | 1.609 | 0.011 |
|  | $\bar{s}^{2}$ | -4.493 | 2.594 | 0.083 |
| **uRBC(ref: uRBC0)** |  |  |  |  |
| uRBC2 | 1 | 1.269 | 0.610 | 0.038 |
|  | $\bar{s}$ | -5.142 | 3.075 | 0.095 |
|  | $\bar{s}^{2}$ | 5.151 | 2.895 | 0.075 |
| **ALB** | 1 | -0.701 | 0.179 | 0.000 |
|  | $\bar{s}$ | 1.496 | 0.530 | 0.005 |
|  | $\bar{s}^{2}$ | -1.01 | 0.604 | 0.094 |
| **PLT** | $\bar{s}$ | -0.344 | 0.109 | 0.002 |
|  | $\bar{s}^{2}$ | 0.258 | 0.147 | 0.079 |
| **Tac** | $\bar{s}$ | 0.422 | 0.228 | 0.064 |
|  | $\bar{s}^{2}$ | -0.733 | 0.355 | 0.039 |
| **NE** | $\bar{s}$ | 0.250 | 0.119 | 0.036 |
| **LYM** | 1 | 0.058 | 0.032 | 0.072 |
|  | $\bar{s}$ | -0.287 | 0.157 | 0.068 |
|  | $\bar{s}^{2}$ | 0.309 | 0.163 | 0.058 |

Induction, immune induction scheme; ATG, antithymocyte globulin; DGF, delayed graft function; AR, acute rejection; BMI, body mass index; eGFR, estimated glomerular filtration rate; uPRO, urinary protein; uPRO0, negative result of urinary protein; uPRO1, low level of urinary protein; uPRO2, high level of urinary protein; uWBC, urinary leukocyte; uWBC0, negative result of urinary leukocyte; uWBC1, low level of urinary leukocyte; uRBC, urinary erythrocyte; uRBC0, negative result of urinary erythrocyte; uRBC2, high level of urinary erythrocyte; ALB, serum albumin; PLT, platelet count; Tac, blood tacrolimus concentration; NE, blood neutrophil count; LYM, blood lymphocyte count; SE, standard error.
